# The Impact of Digital Literacy on Social Isolation Among Korean American Older Adults: Insights From the PLAN Trial

**DOI:** 10.1093/geroni/igaf122.365

**Published:** 2025-12-31

**Authors:** Deborah Min, Tina Kim, Hae-Ra Han

**Affiliations:** Johns Hopkins Bloomberg School of Public Health, United States; Johns Hopkins Hospital, Baltimore, Maryland, United States; Johns Hopkins University, Baltimore, Maryland, United States

## Abstract

Social isolation is a pressing public health concern both in the United States and globally. While growing research highlights the urgent need to address social isolation to enhance the health and well-being of older adults, the magnitude and nature of this issue among immigrant older adults—especially those with limited English proficiency—is not completely known. Immigrant older adults with limited English proficiency are particularly at risk for social isolation, which may be further exacerbated by low digital literacy and engagement. This multi-method study examines the relationship between digital literacy and social isolation using quantitative data from Korean American older adults (N = 733) screened for the PLAN trial (Preparing healthy aging through dementia Literacy education And Navigation; ClinicalTrials.gov Identifier: NCT03909347). The quantitative sample had a mean age of 77 years, with 70% female, 58% having a high school education or less, 28% reporting comfort with technology, and 37% identified as at risk for social isolation. To contextualize our understanding of these findings, we are currently conducting qualitative interviews with a subset of participants to explore the lived experiences of social isolation and the role of digital literacy among community-dwelling Korean American older adults. These combined insights will serve as a critical first step in informing culturally-responsive intervention strategies aimed at reducing social isolation and improving the well-being of immigrant older adults with limited English proficiency in the United States.

